# Common Arterial Trunk Repair at the Red Cross War Memorial Hospital, Cape Town: A 20-Year Review of Surgical Practice and Outcomes

**DOI:** 10.1177/21501351241256582

**Published:** 2024-07-23

**Authors:** A Moodley, HM Meyer, S Salie, P Human, L J Zühlke, A Brooks

**Affiliations:** 1 Division of Cardio-Thoracic Surgery, 37716 University of Cape Town, Cape Town, South Africa; 2 Division of Paediatric Anaesthesia, Department of Anesthesia and Perioperative Medicine, Faculty of Health Sciences, 37716 University of Cape Town, Cape Town, South Africa; 3 Division of Paediatric Critical Care, School of Child and Adolescent Health, University of Cape Town, Cape Town, South Africa; 4 Paediatric Intensive Care Unit, Red Cross War Memorial Children's Hospital, Cape Town, South Africa; 5 South African Medical Research Council, Francie van Zijl Drive, Cape Town, South Africa; 6 Division of Paediatric Cardiology, Department of Paediatrics and Child Health, Faculty of Health Sciences, University of Cape Town, Red Cross War Memorial Children's Hospital, Cape Town, South Africa

**Keywords:** truncus arteriosus surgery, aortopulmonary septal defect, persistent truncus arteriosus, cardiac septal defects, congenital heart defect, cardiovascular abnormality, cardiovascular disease

## Abstract

**Background:**

This study describes the 20-year experience of managing common arterial trunk (CAT) in a low-and-middle-income country and compares the early and medium-term outcomes following the transition from conduit to nonconduit repair at the Red Cross War Memorial Children's Hospital.

**Methods:**

Single-center retrospective study of consecutive patients aged less than 18 years who underwent repair of CAT from January 1999 to December 2018 at the Red Cross War Memorial Children's Hospital. Patients with interrupted aortic arch or previous pulmonary artery banding were excluded.

**Results:**

Fifty-four patients had CAT repair during the study period. Thirty-four (63.0%) patients had a conduit repair, and 20 (37.0%) patients had a nonconduit repair. There were two intraoperative deaths. Thirty-day in-hospital mortality was 22.2% (12/54). Overall, in-hospital mortality was 29.6% (16/54). Eight (21.1%) late mortalities were observed. The actuarial survival for the conduit group was 77.5%, 53.4%, and 44.5% at 6, 12, and 27 months, respectively, and the nonconduit group was 58.6% at six months. The overall freedom from reoperation between the conduit group and nonconduit group was 66.2% versus 86.5%, 66.2% versus 76.9%, and 29.8% versus 64.1% at 1, 2, and 8 years, respectively.

**Conclusions:**

The outcomes following the transition to nonconduit repair for CAT in a low- and middle-income setting appear to be encouraging. There was no difference in mortality between conduit and nonconduit repairs, and importantly the results suggest a trend toward lower reintervention rates.

## Introduction

Common arterial trunk (CAT) is a rare congenital cardiac condition, accounting for less than 3% of all critical congenital cardiac heart anomalies.^1^^,2^ Without surgical intervention, the defect has a mortality rate of 70% to 85% within the first year of life.^3-5^ In 1967, McGoon and Rastelli pioneered CAT repair using an aortic homograft.^6^ In the 1990s, Barbero-Marcial et al described a nonconduit method using the left atrial appendage and autologous pericardium to reconstruct the neo-main pulmonary artery (PA).

Data on outcomes following CAT repair remain primarily derived from high-income countries (HICs).^3,8-17^ Despite improvements in perioperative care, perioperative mortality remains significant. Risk factors for poor outcomes include low birth weight, coronary artery issues, prolonged aortic cross-clamp time, interrupted aortic arch (IAA), and truncal valve regurgitation.^3,8,9,14^ Data on outcomes after CAT repair in low- and middle-income countries (LMICs)^18^ are scarce.^19-21^ and published data on outcomes after CAT repair in Africa are limited to a single case report.^22^

The debate over conduit versus nonconduit repair in CAT surgery persists.^15^ Although in-hospital morbidity and mortality appear similar,^23^ nonconduit repair may spare the patient from multiple reoperations as the child grows, particularly valuable in resource-limited settings. Our institution has been performing CAT repair since the 1990s, and in 2007, a decision was made to transition to nonconduit repair for CAT. This was due to global homograft shortages and concerns regarding implantation and longevity with the alternative “off-the-shelf” conduits such as the Contegra and Hancock grafts, as well as the aim of reducing surgical reinterventions. Another major challenge in LMICs is delayed diagnosis and presentation, which further complicates the management of children with CAT presenting for surgery.

We hypothesized that the move toward nonconduit repair would lower reintervention rates in our patients without significant adverse outcomes. Our objective was to describe postoperative outcomes following CAT repair over a 20-year period prior to and following the transition to nonconduit repair at our institution.

The primary aim was to describe the institutional experience in managing CAT at Red Cross War Memorial Children's Hospital over a 20-year period. The secondary aim was to compare the early and medium-term outcomes following transition from conduit to nonconduit repair.

## Materials and Methods

### Study Population

Single-center retrospective study of all consecutive patients who underwent the repair of CAT from January 1999 to December 2018 at Red Cross War Memorial Children's Hospital. Patients with IAA and those who had a previous PA banding procedure were excluded from the study.

Ethical consent for the study was given by the Human Research Ethics Committee of the Faculty of Health Sciences, University of Cape Town (HREC REF: R017/2014, 044/2016 & 655/2018). Local hospital authorization was approved by the Research Review Committee. Specific patients were de-identified, and the need for obtaining formal consent for retrospective analysis of data from patients or their caregivers was waived.

### Data Collection

Relevant data were collected from the pediatric cardiac surgery database, pediatric cardiology database, pediatric intensive care unit (PICU) records, and matching patient folders. Attempts were made to contact the parent or legal guardian of every patient who had been lost to follow-up.

Patients were divided in two groups, group 1 representing the conduit reconstruction method and group 2 representing the nonconduit group of reconstruction.

Patient demographics, intraoperative, and postoperative data included length of general anesthesia, surgical operative duration, total cardiopulmonary bypass (CPB) time, aortic cross-clamp time, concurrent procedures, conduit utilization, PICU length of stay (PICU LOS), and hospital LOS (HLOS) data were collected.

Data on echo findings were recorded at three time points: preoperative, early postoperative (<48 h postop) and most recent.

Complications included unplanned cardiac catheterization, unscheduled cardiac reoperation (not including reoperation for bleeding), bleeding necessitating reoperation, acute kidney injury defined using the Kidney Disease Improving Global Outcomes (KDIGO) criteria, infective complications, neurologic consequences persisting at discharge, noninfective pulmonary complications (failure to extubate, pneumothorax, pleural effusion, chylothorax, or diaphragmatic paresis), myocardial arrest during or after the procedure, and death as well as any other nonpredefined complications. Infective complications included bacteriemia, lower respiratory tract infection (LRTI)—confirmed by chest x-rays with new or progressive and persistent pathological infiltrates—consolidation, cavitation, clinical diagnosis, superficial, or deep sternal wound infection or any other type of infection (defined as a confirmed diagnosis of verified or assumed sepsis by the treating pediatricians).

“Early mortality” was defined as death occurring within 30 days of the operation or before hospital discharge. Death after this time was categorized as “late mortality.”

Medium- to long-term records composed of years to reintervention, late mortality, and patients lost to follow-up (defined as no outpatient or inpatient appearance for the extent of 24 months).

## Operative Technique

The surgical repair was performed following a full median sternotomy incision and with CPB support in all patients under moderate to deep hypothermic conditions. The PAs were ensnared at the commencement of CPB, and the heart was arrested with antegrade cardioplegia. The PAs were excised from the truncal vessel while taking care to prevent damage to the truncal valve and the coronary arteries. The aortic root was repaired using bovine pericardium.

Pulmonary arteries were extensively mobilized in all cases. Inspection of the truncal valve for morphology and function was conducted, and truncal valve regurgitation repair was effected where required. The ventricular septal defect (VSD) was almost always closed through the ventriculotomy. In group 1, right ventricle to PA (RV-PA) continuity was established using a conduit. In group 2, the pulmonary outflow tract was recreated utilizing the left atrial appendage, which was sutured to the distal end of the right ventriculotomy and the pulmonary convergence, maintaining its anatomical position. The anterior hood was reconstructed with a bovine pericardial patch. The sternum was electively left open, and delayed closure was performed in the PICU.

### Statistical Analysis

Analysis was performed using JMP Pro (version 16.0, SAS). Normality of continuous numerical variable distribution was determined by Shapiro-Wilk analysis. Normally distributed continuous numerical variables were expressed as means ± standard deviation, with inferential analysis performed using the independent Student *t* test. Non-normally distributed variables were expressed as medians ± range with between-group comparisons performed using the Wilcoxon test. Analysis of categorical variables was assessed using Fisher exact or χ^2^ testing. Evaluations of time-related survival and freedom from reintervention were determined using the Kaplan-Meier method with reported log-rank and Wilcoxon rank-sum between-group testing. Two-tailed significance levels <.05 were accepted to define statistical significance.

## Results

### Patient Characteristics

Fifty-four patients had CAT repair during the study period (Table 1). The median age at the time of surgery was 61.5 days [IQR 44.8-138.8 days], and the median weight was 3.4 kg [IQR 3.0-4.5 kg]. Genetic syndromes were present in 17 (31.5%) cases. These included 16 (29.7%) patients with Di George syndrome (22q11.2 deletion syndrome) and one patient with VACTERL syndrome.

**Table 1.** Conduit Versus Nonconduit.

Variable	All patients (n = 54)	Group 1 (Conduit) n = 34	Group 2 (Nonconduit) n = 20	Chi-square *P* value
Age (days), median (IQR)	61.5(44.8-138.8)	90.5(58-163.5)	40.5(28-59)	<0.0001^a^
Weight (kg), median (IQR)	3.4 (3.0-4.5)	3.9(3.3-5.0)	3.1(2.7-3.4)	0.0002^a^
Sex, n (%)				
Female	27 (50.0)	16(47.1)	11(55.0)	0.573^b^
Duration of CPB (mins), median (IQR)	163(140-199)	143(130-162)	196.5(172-223.8)	<0.0001^a^
Ischaemic time (mins), median (IQR)	104(89-126)	91.0(82-104)	122.5(112.3-148)	<0.0001^a^
Open sternum, n (%)	32 (59.3)	15(44.1)	17(85.0)	0.003^b^
Duration of mechanical ventilation (days), median (IQR)	7(2-12)	7(4-12)	6(1.3-8.0)	0.202^a^
ICU LOS (days), median (IQR)	10(6-20)	10(7-22)	10(5.3-14.3)	0.445^a^
Postoperative complications, n (%)	29(53.7)	19(55.9)	10(50.0)	0.675^b^
Hospital LOS (days), median (IQR)	30(18-53)	33(20-63)	25(17.3-36.8)	0.086^a^
Reinterventions, n (%)	13(24.1)	10(29.4)	3(15.0)	0.329^b^
Late deaths, n (%)	8(14.8)	7 (20.6)	1 (5.0)	0.721^b^

Abbreviations: CPB, cardiopulmonary bypass; ICU, intensive care unit; IQR, interquartile range; LOS, length of stay

^a^ Wilcoxon test.

^b^ Fisher exact test.

Thirty-four (63.0%) patients had a conduit repair, and 20 (37.0%) patients had a nonconduit repair. Patients who underwent a conduit repair were significantly older compared with the patients who underwent nonconduit repair. The median total perfusion time and median ischemic time were significantly lower in the conduit group compared with the nonconduit group (*P *< .0001) (Table 1). An aortic homograft was used in 31 of 34 (91%) of conduit cases. A Contegra^R^ graft was used in two patients. There was no significant difference in the duration of mechanical ventilatory support, duration of ICU stays, or HLOS between the group who underwent conduit versus nonconduit repair (Table 1).

### In-Hospital Mortality

Thirty-day in-hospital mortality was 22.2% (12/54), and overall in-hospital mortality was 29.6% (16/54).. This included two on-table deaths. The first patient, with CAT Type 1, presented for surgery at 22 days of age and was unable to be weaned off CPB due to right heart failure secondary to suspected pulmonary hypertension. The second patient (44 days of age), with CAT type 2, died due to technical complications associated with abnormal coronary anatomy.

Thirteen (81.3%) of 16 deaths occurred in the PICU. The causes of death included 8 of 13 (61.2%) low cardiac output syndrome (LCOS), 1 of 13 (7.7%) pulmonary hypertensive crisis, and 4 of 13 (30.7%) secondary to septic complications, including one patient with methicillin-resistant *Staphylococcus aureus* (MRSA) infective endocarditis. Onedeath occurred in the ward after ICU discharge on postoperative day 40, secondary to aspiration pneumonia. There was no difference in mortality rates between those patients who underwent a conduit repair versus those patients who underwent a nonconduit repair (*P *= .507). There was no significant difference in mortality between the group of patients whose sternums were closed versus those with delayed sternal closure.

### Morbidity

Among the 52 patients, 29 (55.8%) experienced postoperative complications (Table 2). No significant difference in complication rates existed between those who underwent conduit versus nonconduit repair (*P* = .675). In the entire cohort, 12 of 54 (22.2%) patients suffered multiple complications. The most common complications were cardiovascular and infection. Low cardiac output syndrome rates were similar in the conduit (6/34, 17.6%) and nonconduit (4/20, 20%) groups. Infectious complications affected 13 of 52 (25%) patients, with comparable rates in both groups (*P* = 1.000). Positive microbiology was noted in 12 of these 13 patients, including *Klebsiella pneumonia* (n = 3), viral pneumonia (n = 3), *Serratia marcescens* (n = 1), and *Sphingomonas paucimobilis* (n = 1). The primary septic complication was LRTI (9/13, 69.2%), while two patients had superficial wound sepsis. In the nonconduit group, MRSA was cultured from one patient's sternal wound, necessitating debridement and secondary closure. In the conduit group, *Staphylococcus epidermidis* sensitive to Cloxacillin was isolated, managed with three vacuum-assisted dressings and secondary closure.

**Table 2.** Postoperative Complications.

Complication	Total number of complications n, (%)	Conduit (n, %)	Nonconduit (n, %)	*P* value
**Infective**	**13** (**24.1)**	**8** (**23.5)**	**5** (**25.0)**	**1**.**0^a^**
Lower respiratory tract infection	9 (16.6)	5 (14.7)	4 (20.0)	
Wound sepsis	2 (3.7)	1 (2.9)	1 (5.0)	
Bloodstream	1 (1.9)	1 (2.9)	0	
Urinary tract infection	1 (1.9)	1 (2.9)	0	
**Cardiac reoperation**	**5** (**9.3)**	**3** (**8.8%)**	**2** (**10.0%)**	**1**.**0^a^**
Bleeding	2 (3.7)	2 (5.9)	0	
Homograft dilation and RPA compression	1 (1.9)	1 (2.9)	0	
VSD patch leak	1 (1.9)	0	1 (5.0%)	
Branch pulmonary artery stenosis	1 (1.9)	0	1 (5.0%)	
**Cardiac**	**13** (**24.1)**	**8** (**23.5)**	**5** (**25.0)**	**0**.**903^b^**
Low cardiac output syndrome	10 (18.5)	6 (17.6)	4 (20.0)	
Pericardial Effusions	3 (5.6)	2 (5.9)	1 (5.0)	
**Other pulmonary**	**8** (**14.8)**	**4** (**11.8)**	**4** (**20.0)**	**0**.**450^a^**
Chylothorax	1 (1.9)	1 (2.9)	0	
Pleural effusion	6 (11.1)	3 (8.8)	3 (15.0)	
Phrenic nerve palsy	1 (1.9)	0	1 (5.0)	
**Acute kidney injury**	6 (11.1)	5 (14.7)	1 (5.0)	**0.395^a^**
**Total**	**29,** (**53.7%)**	**19,** (**55.9%)**	**10,** (**50.0%)**	**0**.**675^b^**

Abbreviations:RPA, right pulmonary artery; VSD, ventricular septal defect;

^a^ Fisher exact test.

^b^ Chi-square.

Three unplanned cardiac reoperations occurred before discharge: one in the conduit group, involving homograft replacement and relief of right PA compression; two in the nonconduit group, addressing a VSD patch leak and branch PA stenosis. In the conduit group, two patients needed reoperation for bleeding; one due to cardiac tamponade and the other for bleeding around the conduit anastomotic site. Of the 18 patients with preoperative moderate truncal valve regurgitation, nine (50%) patients had residual moderate postoperative truncal valve regurgitation. Truncal valve regurgitation in all four patients who presented with “severe” preoperative truncal valve regurgitation had improved to “mild” postoperatively.

### Medium-Term Outcomes

A total of 38 cases were followed up post hospital discharge for a median of 23 months (IQR 5.5-89.3 months). Eight (21.1%) late mortalities were observed (Table 3). The mean time to death post discharge was 9.4 months (SD+/- 9.6) for the conduit group. Most late deaths occurred in the conduit group (7/8, 87.5%). Within the conduit group, the causes included lower respiratory tract infection(LTRI) in four patients, of which one was secondary to post-procedural compression of the left main bronchus by the extracardiac conduit. Of the remaining patients, one patient died of sepsis post-Nissen fundoplication, one patient died due to massive hemoptysis secondary to an esophageal subclavian fistula and in one patient the cause of death was unknown. The single late mortality observed in the nonconduit group occurred at six months and was due to hypoxic cardiac arrest secondary to recurrent LRTI.

**Table 3.** Medium-Term Outcomes Post Hospital Discharge.

	Whole cohort N = 38	Conduit N = 25	Nonconduit N = 13	*P* value
Lost to follow up, n (%)				
	11 (28.9%)	8 (32.0%)	3 (23.1%)	0.46^a^
Mortality, n (%)				
	8 (21.1%)	7 (28.0%)	1 (7.7%)	0.22^a^
Actuarial survival rate (%)				
6 months	62.1	77.5	58.6	0.47^b^
12 months	55.8	53.4	58.6	0.60^b^
27 months	50.6	44.5	58.6	0.97^b^
Reintervention, n (%)				
	10 (26.3%)	9 (36.0%)	1 (7.7%)	0.12^a^
Overall freedom from reoperation (%)				
1 year	74.0%	66.2%	86.5%	0.52^b^
2 years	70.1%	66.2%	76.9%	0.22^b^
8 years	38.8%	29.8%	64.1%	0.24^b^

^a^ Fisher exact test.

^b^ Wilcoxon test.

The actuarial survival rate for the entire cohort was 62.1%, 55.8%, and 50.6% at 6 months, 12 months, and 27 months, respectively (Table 3). Eleven (28.9%) of 38 cases were lost to follow-up.

### Reintervention

During the study period, 10 (26.3%) patients required further intervention post hospital discharge (Table 3). Within the group who underwent a conduit repair, the indications for reoperation included RVOT stenosis in 5 of 9 (55.6%) patients, VSD patch leak (1/9, 11.1%), VSD patch dehiscence (1/9, 11.1%), endocarditis of the aortic valve and VSD patch (1/9, 11.1%), left thoracotomy for aortopexy which was performed to relieve left main bronchus compression secondary to a dilated aorta on a patient who had a previous CAT repair with a homograft (1/9, 11.1%) and one patient had percutaneous balloon dilation for branch PA stenosis six months post-CAT repair. Within the group that underwent a nonconduit repair, one patient required a homograft due to RVOT stenosis.

The overall freedom from reoperation for the patients who underwent a conduit repair was 66.2% versus 86.5%, 66.2% versus 76.9%, and 29.8% versus 64.1% compared with the patients who underwent a nonconduit repair at one, two, and eight years, respectively (Table 3).

**Figure 1.**
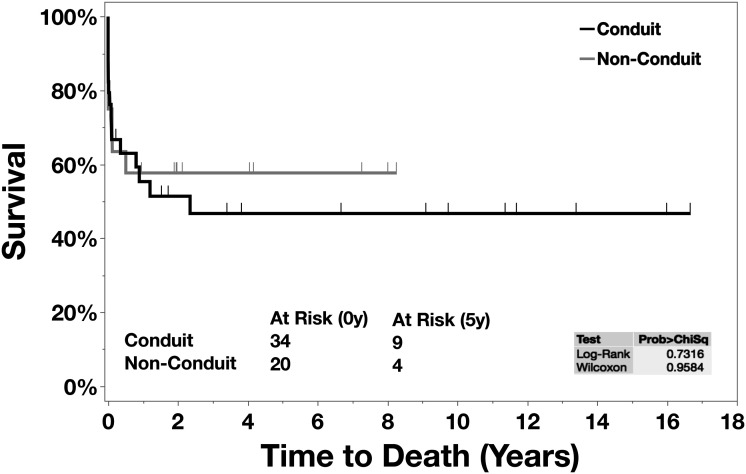
Kaplan-Meier: survival curve.

## Discussion

**Figure 2.**
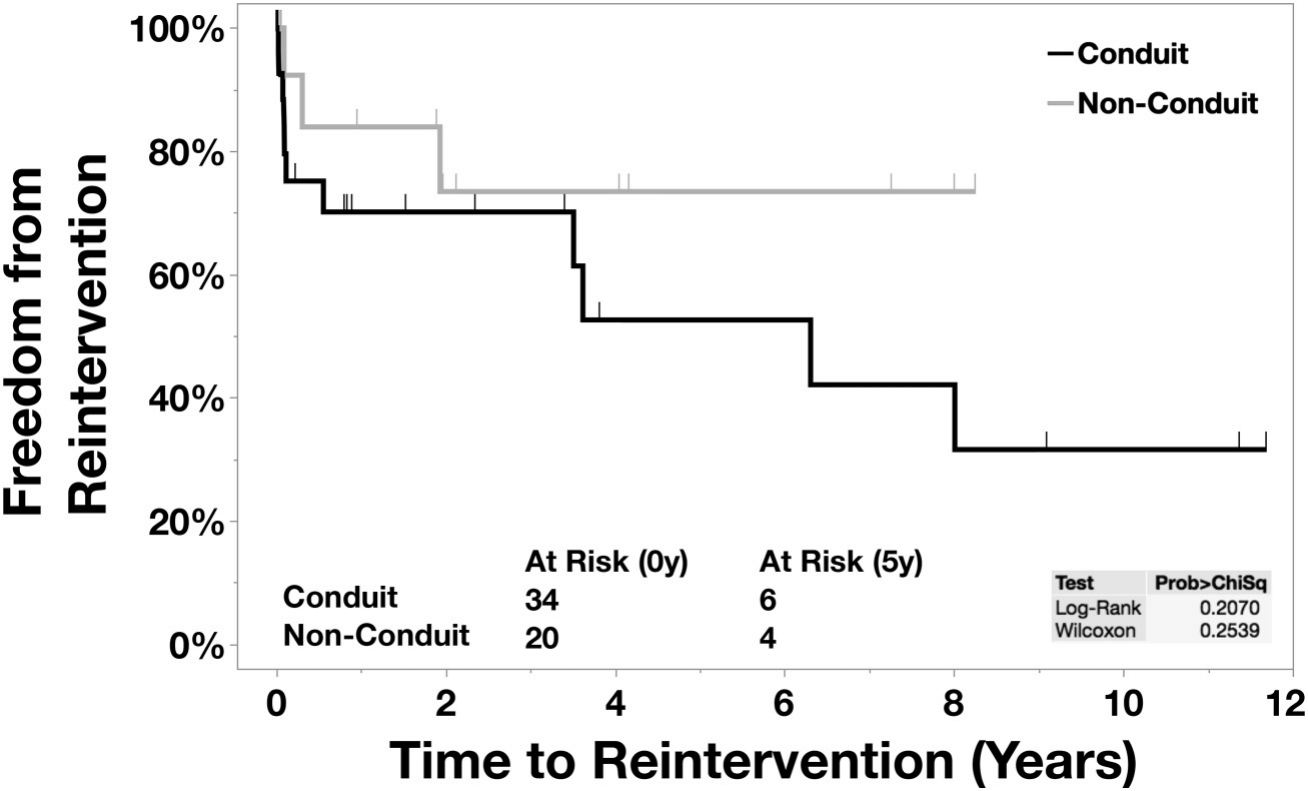
Kaplan-Meier: Reinterventions

The principal finding of this study was that 30-day and in-hospital postoperative mortality following CAT repair at our institution during a 20-year period was 22.2% and 29.6%, respectively. There was no difference in perioperative mortality between the groups that either underwent a conduit or a nonconduit repair (Figure 1). The overall freedom from reoperation was markedly lower at eight years in the nonconduit group (Figure 2).

Data published from a single center in Thailand do provide some insight into CAT repair outcomes in LMICs.^20^ Dangrungroj et al reported on outcomes following CAT repair in 74 patients from 1995 to 2018, revealing a 30.8% early mortality rate, which closely resembled our findings.^21^ Early mortalities were often linked to pulmonary hypertensive crises, resulting in LCOS. In our study, LCOS, frequently associated with pulmonary hypertensive crises, was also a critical factor in early mortality, accounting for 62.4% of perioperative deaths. Additionally, septic complications played a significant role in early mortality, with rates of 31.3% in our study and 25.0% in Dangrungroj's study.

In comparison to our study and Dangrungroj et al findings, HICs report both younger age the time of surgery and lower perioperative mortality rates (5%-17%) following CAT repair^.13,16,23^ Initially, we operated on CAT patients at an older age, mainly in the first decade for the study period, due to limited conduit sizes. Late postnatal diagnosis was also common due to limited prenatal ultrasound access. In recent years, improvements in equipment and expertise have facilitated earlier postnatal diagnoses enabling us the opportunity to operate earlier. While our strategy has moved primarily toward nonconduit repair, we still consider valved conduit repair in select cases with severe pulmonary hypertension. Factors contributing to the relatively higher perioperative mortality seen in LMICs are poorly understood, but likely include prolonged hospital stays preoperatively, delayed diagnosis with prolonged pulmonary overcirculation and recurrent LRTIs. The older age at time of surgery in our cohort may also be associated with increased postoperative mortality risks. It has been well-documented that surgery beyond the neonatal period is associated with increased postoperative mortality rates, commonly related to pulmonary hypertensive crises and LCOS, the leading cause of in-hospital death in our cohort.^16,23^

Multiple studies in HICs have highlighted preoperative risk factors linked to early mortality in CAT repair, including coronary abnormalities, severe truncal valve regurgitation, and PA issues.^11-13,17^ In our cohort, six patients had abnormal coronary anatomy, and one patient with such anatomy died due to surgical complications. Severe preoperative truncal valve regurgitation was observed in one patient who survived and remained alive during follow-up. While data on the association between longer duration of CPB and mortality are conflicting, our study found no significant difference in mortality despite longer CPB and ischemic times in the nonconduit repair group. It is, however, important to note that CPB strategies and myocardial protection have undergone significant changes over time. It is possible that these changes have offset the negative effect of longer bypass and cross-clamp times, and these markers almost certainly remain significant risk factors. Univariate analysis did not identify specific risk factors for perioperative mortality in our cohort.

### Medium-Term Outcomes

Thirty-eight patients survived to hospital discharge, but eight experienced late deaths, primarily due to LRTI, mirroring findings by Dangrungroj et al. Concerningly, nearly 29% of discharged patients were lost to follow-up, a trend seen in our previous bidirectional Glenn surgery outcomes study.^24^ as well as our study on the efficacy of PA banding.^25^ The high incidence of lost to follow-up is consistent throughout our studies, and this factor needs to be considered when a treatment strategy is chosen. Factors contributing to a high incidence of lost to follow-up include the misconception of surgery as a cure, limited access to transportation, financial constraints for rural patients, scarcity of specialized pediatric cardiac centers, and frequently changing contact information. Notably, lost to follow-up is especially concerning for conduit repair patients, as reintervention needs increase over time. In 2009, Raisky et al proposed the nonconduit technique as an alternative to aortic homografts,^23^^14^ noting similar perioperative morbidity and mortality between the two techniques but noted a marked reduction in the need for reintervention in the nonconduit group. The authors highlighted the lack of aortic homografts, necessitating a trend toward performing a nonconduit technique or using heterografts to establish RV-PA continuity.^23^ While many of our conduit repair patients received aortic homografts due to earlier availability, our results align with Raisky et al, showing improved freedom from reoperation at eight years in the nonconduit group. This emphasizes the potential benefits of nonconduit techniques in reducing the need for reintervention.

### Suggestions for Improvement

Early detection and referral are crucial for improved CAT patient outcomes. Ideally, CAT should be identified through prenatal or early postnatal screening. While current guidelines recommend that all pregnant women should receive one basic obstetric ultrasound at 18 to 20 weeks’ gestation,^26^ ultrasound is a relatively expensive operator-dependent resource-intensive tool, and the implementation of universal fetal anomaly screening is not currently realistic within the public healthcare setting in South Africa. A practical alternative is screening for congenital cardiac issues using peripheral oxygen saturation measurement during routine postnatal examinations. This noninvasive, cost-effective, highly specific, and moderately sensitive test is standard in many HICs^27^. However, limited staffing and infrastructure have hindered universal peripheral oxygen saturation screening for neonates in the South African context.^28,29^

Even if strides toward earlier diagnosis of CAT are made, limitations in the provision of pediatric cardiac surgical services remain. Initiatives to improve and reduce the cases of pediatric patients lost to follow-up are also already in progress. Children who underwent surgery are actively followed up within 30 days of surgery as part of the International Quality Improvement Collaborative for Congenital Heart Disease.^19^

## Limitations

This study has some key limitations. These findings are retrospective and represent outcomes from a single center from a relatively small cohort of patients. Implications from this study and generalizability should be deduced within this context.

When comparing the two groups, it is also important to acknowledge several confounding factors that complicate this comparison. Over the 20-year study period, not only did surgical techniques evolve but also CPB techniques and methods of myocardial protection were not consistent. The group that underwent conduit repair tended to be older at the time of surgery and were operated on during an earlier era, by different surgeons. During the era of conduit repair, the practice was to wait until patients were suitable for an adequately sized conduit. It is probable that there was attrition of smaller, sicker patients, leading to a self-selection process where only patients who survived the early period with CAT underwent surgery.

Additionally, it is important to note that the decision to leave the sternum open post-complex neonatal cases or in younger or smaller children has represented a deliberate shift in practice implemented since 2007. The high incidence of open sternums is primarily an elective decision, which the authors believe offsets many of the early complications in our setting. Given this context, drawing comparisons between the two groups; closed sternums versus open sternums, poses a challenge.

The nonconduit technique was also implemented more recently, and the interval mortality and requirement for reintervention in our study cohort should be interpreted with caution given the substantial number of cases lost to follow-up and the shorter follow-up period in the nonconduit group. However, the lost to follow-up rates is reflective of contextual challenges in LMICs and is also reflective of similar experiences noted in our institution and in other centers in Africa.^19^

## Summary

This is the first study reporting on a series of patients undergoing CAT repair in Africa. Important findings are the older age at which the surgery is carried out compared with HICs, similar mortality rates to other LMICs, reduced need for reintervention in the nonconduit group and the number of patients lost to follow-up. The findings of this study support the decision to transition to a nonconduit repair in our situation but emphasize the need for more studies to identify valuable strategies to improve early diagnosis of CAT, expedite surgery, and reduce the number of patients lost to follow-up.

## Abbreviations

CAT: common arterial trunk
CPB: cardiopulmonary bypass
HICs: high-income countries
HLOS: hospital length of stay
LCOS: low cardiac output syndrome
LMICs: low- and middle-income countries
LRTI: lower respiratory tract infection
PAs: pulmonary arteries
PICU: pediatric intensive care unit
PICU LOS: pediatric intensive care unit length of stay
RCWMCH: Red Cross War Memorial Children's Hospital
RPA: right pulmonary artery
RVOT: right ventricular outflow tract
RV-PA: right ventricle to pulmonary artery
VSD: ventricular septal defect
